# Commentary on: Wong YL, Wong SW, Ting DSJ, Muralidhar A, Sen S, Schaff O, et al. Impacts of climate change on ocular health: A scoping review. J Clim Chang Heal. 2024 Jan 1;15:100296. doi: 10.1016/j.joclim.2023.100296.

**DOI:** 10.1016/j.joclim.2025.100441

**Published:** 2025-03-18

**Authors:** Seth Dhillon, Baljean Dhillon

**Affiliations:** a Ophthalmology Specialty Trainee Year 2, The Princess Alexandra Eye Pavilion, Edinburgh, UK; b Centre for Clinical Brain Sciences, School of Clinical Sciences, College of Medicine and Veterinary Medicine, University of Edinburgh, Edinburgh, UK

We read with interest the very timely and comprehensive review by Wong et al. [1]. Since the publication of this scoping review, new evidence regarding microplastics (MPs) has emerged, offering further insights into their impact on ocular health in the context of climate change. This new information may be of interest to readers and emphasizes the pressing need for delivering interdisciplinary research in this rapidly evolving field.

MPs, defined as plastic particles smaller than 5 mm, represent a growing global concern and are closely linked to climate change [2]. Primary MPs are intentionally manufactured to a small size and secondary MPs result from the fragmentation of larger plastic items [3].

Plastic production not only relies on fossil fuels and drives greenhouse gas emissions, but microplastics themselves also contribute to climate change. They disrupt carbon sequestration by affecting phytoplankton and zooplankton, accumulate in ice caps—accelerating their melting—and release greenhouse gases as they degrade [2].

These particles have become ubiquitous in our environment and have been shown to accumulate in human tissues via ingestion and inhalation [3,4]. Nihart et al. reported an increasing trend of micro and nanoplastics (MNPs) found in the brain and liver, with brain samples from dementia decedents exhibiting greater MNP presence [5].

Zhong et al. identified and quantified the specific types of MPs within 49 vitreous samples. Correlations were found between MP levels and ocular health parameters including intraocular pressure and the presence of aqueous humour opacities. Individuals with retinopathy exhibited a greater MP-associated ocular health risk [6]. Li et al. demonstrated the toxic effects of nanoplastics on retinal pigment epithelial cells [7].

MPs promote chronic inflammation, oxidative stress and changes in homeostasis, as demonstrated in experimental models [8]. With further exploration via observational and tissue-based studies, we will gain a clearer understanding of the impact of microplastics on ocular health.

## CRediT authorship contribution statement

**Seth Dhillon:** Conceptualization, Data curation, Formal analysis, Methodology, Writing – original draft, Writing – review & editing. **Baljean Dhillon:** Supervision, Writing – review & editing.

## Declaration of competing interest

The authors declare that they have no known competing financial interests or personal relationships that could have appeared to influence the work reported in this paper.
